# Alteration of fronto-thalamic-striatal and visual network activity to positive emotional stimuli in adolescent patients with bipolar disorder during a Go/No-Go task-based functional brain MRI

**DOI:** 10.1016/j.nicl.2026.104005

**Published:** 2026-05-10

**Authors:** Xueying Wang, Jinfan Zhang, Feifei Wu, Liying Shen, Lin Wang, Han Wu, Qian Xiao, Xiaoping Yi, Xiaoqun Liu, Alessandro Grecucci, Bihong T. Chen

**Affiliations:** a Department of Nuclear Medicine and Department of Radiology, Chongqing University Three Gorges Hospital, Chongqing University, Chongqing 404000 Chongqing, PR China; b Department of Radiology, Xiangya Hospital, Central South University, Changsha 410008, Hunan, PR China; c Mental Health Center of Xiangya Hospital, Central South University, Changsha 410008, Hunan, PR China; d Department of International Health, Graduate School of Medicine, The University of Tokyo, Tokyo, Japan; e National Clinical Research Center for Geriatric Disorder (Xiangya Hospital), Central South University, Changsha 410008, Hunan, PR China; f National Engineering Research Center for Personalized Diagnostic and Therapeutic Technology, Xiangya Hospital, Changsha 410008, Hunan, PR China; g Medical Imaging Center (MIC), Clinical Research Center (CRC), Medical Pathology Center (MPC), Cancer Early Detection and Treatment Center (CEDTC) and Translational Medicine Research Center (TMRC), Chongqing University Three Gorges Hospital, Chongqing University, Chongqing 404000, PR China; h School of Medicine, Chongqing University, Chongqing 400030 Chongqing, PR China; i FuRong Laboratory, Changsha 410078, Hunan, PR China; j Department of Maternal and Child Health, Xiangya School of Public Health, Central South University, Tongzipo Road, Changsha, Hunan 410013, PR China; k Department of Psychology and Cognitive Sciences (DiPSCo), University of Trento, Rovereto (TN) 38068, Italy; l Department of Diagnostic Radiology, City of Hope National Medical Center, Duarte, California 91010, USA

**Keywords:** positive emotional stimuli, adolescents, bipolar disorder, Go/No-Go task, functional brain MRI

## Abstract

**Background:**

Adolescent patients with bipolar disorder (BD) tend to have abnormal neural activity to emotional stimuli. This study aimed to assess alterations of neural activity within the fronto-thalamic-striatal circuit, and the fusiform gyrus during positive stimuli processing in Go/No-Go task-based brain functional MRI (fMRI).

**Methods:**

This prospective study enrolled 43 adolescent patients with BD and 18 age- sex-matched healthy controls. All study participants underwent a task-based brain fMRI using a happy versus neutral Go/No-Go paradigm. All study participants also completed multiple affective and cognitive assessment questionnaire.

**Results:**

Enhanced activity was found in the fronto-thalamic-striatal circuit including the inferior frontal gyrus and the caudate, the fusiform gyrus, the left cerebellum crus I and the hippocampus in adolescent patients with BD, during response inhibition to happy versus neutral distractors in an emotional Go/No-Go fMRI task, compared with matched healthy controls (*p*<0.05). Moreover, the left inferior frontal gyrus, the right fusiform gyrus and the left cerebellum crus I responses to happy versus neutral distractors were positively associated with the differences in false response errors in the patients with BD (all *p*<0.05, FDR corrected). The enhanced activity of the caudate nucleus and that of the right hippocampus were negatively correlated with cognitive function (all *p*<0.05, FDR corrected).

**Conclusion:**

This study found significant brain functional alterations in the limbic system, the visual brain network and cerebellum, especially the fronto-thalamic-striatal track and fusiform gyrus, which was correlated with cognitive dysfunction in adolescent patients with BD. These changes may serve as potential neuroimaging correlates of BD in adolescent patients.

## Highlights

• Adolescents with BD showed hyperactivation in fronto-thalamic-striatal and fusiform circuits during emotion inhibition.
• Positive stimuli elicit impaired emotion regulation and response inhibition in adolescents with BD.
• Our findings offer insights into emotion dysregulation and inform targeted therapeutic strategies.

## 1 Introduction

Bipolar disorder (BD) is characterized by recurrent episodes of depression and mania, often leading to an imbalance of emotional homeostasis (Müller and Leweke, 2016). Compared to adults with BD, patients with adolescent-onset BD have more acute onset, a tendency to chronic course, poorer prognosis, and a higher incidence of suicide and substance abuse behaviors (McClellan and Werry, 1997). One of the core symptoms of BD is emotional regulation difficulty. Patients may have a heightened sensitivity to negative stimuli and their impaired ability to regulate negative emotions may create a feedback loop, exacerbating emotional instability and mood fluctuations (Hirshfeld-Becker, 2003). Adolescents with BD may also have notable cognitive impairment, which is linked to challenges in tasks involving emotional regulation (Lawlor-Savage et al., 2014). The cognitive deficits observed in this group especially when negative emotions are used as target encompass a range of domains, including overall cognitive functioning, visuospatial and verbal memory, executive functions such as response inhibition, cognitive flexibility, and working memory (Frías et al., 2014, Elias, 2017).

The role of positive emotions (e.g., positive affect, reward responsiveness) in the etiology of BD has not been thoroughly assessed. Nevertheless, positive emotional cues are more closely related to real-life contexts, such as social interactions, learning, and reward signals in adolescents (Crone and Dahl, 2012, Galvan, 2006, Somerville and Casey, 2010), which are particularly important for this age group and are closely linked to the core symptoms of BD. More importantly, adolescents with BD may exhibit a heightened tendency to lose control when exposed to stimuli that elicit overexcitement or overinvolvement, likely stemming from underlying dysregulation in emotional and cognitive processing systems (Hafeman, 2014). Therefore, focusing on positive emotions in assessment of BD may help to understand the interference effects of specific positive emotions including the associated changes in response inhibition and cognitive control. Furthermore, this approach may also contribute to targeted therapeutic intervention and help to prepare these vulnerable adolescent patients with effective tools to manage their heightened arousal.

Functional magnetic resonance imaging (fMRI) is a non-invasive brain imaging technique that utilizes blood oxygen level-dependent (BOLD) signals to reflect indirect changes in neural activity (Logothetis, 2001). Prior fMRI studies on adult patients with BD have revealed abnormal activity in the neural circuits for cognitive-emotional regulation, implicating an altered fronto-thalamic-striatal circuit (Breakspear, 2015, Delvecchio et al., 2013, Mesbah, 2023, Strakowski, 2012). For instance, adults with BD display increased activity in the amygdala, hippocampus, inferior frontal gyrus, thalamus and basal ganglia during emotional processing as compared to the controls (Mesbah, 2023). In addition, neural circuit models indicate the abnormal functional connectivity between fronto-limbic regions observed during tasks involving emotional perception, cognitive control, and emotional regulation in patients with BD (Rich, 2008, Ladouceur, 2011, Morris, 2012, Passarotti, 2012). In adolescents with BD, functional connectivity disruptions are primarily associated with depressive symptoms and suicidal thoughts, alongside a decrease in cortical thickness in areas linked to cognitive control, such as the anterior cingulate cortex and middle frontal gyrus (Toma, 2019). The thalamus and basal ganglia, integral to the brain reward system (Haber and Knutson, 2010), also play roles in emotional regulation (Péron, 2017). Impulsivity in patients with BD has been linked to dysfunctions in cortical-limbic circuits (Brown, 2007, Congdon, 2010, Lapomarda, 2021, Lapomarda, 2021), with basal ganglia abnormalities present early in the patients with this disorder (Strakowski et al., 2005, Hwang, 2006).

Neurocognitive studies have not consistently shown distinct advantage of positive over negative stimuli in adolescent patients with BD as compared to the healthy controls (Rich, 2010). Variable findings may stem from factors such as task difficulty, the emotional intensity of stimuli, or the specific cognitive and affective processes engaged, all of which can significantly influence attentional bias during behavioral tasks in adolescents with BD. Nevertheless, literature remains limited on neural function under the interference of positive emotions on inhibitory control in adolescents with BD. The underlying neurobiological mechanism for Adolescents with BD remains unclear.

To fill this gap in knowledge, we performed this prospective study and recruited a group of adolescents with BD and age-and-gender matched healthy controls (HCs). All participants underwent brain fMRI scans under the Go/No-Go task paradigm, cognitive testing, and clinical assessments of emotional dysregulation. In this study, we used an emotional Go/No-Go task paradigm, comparing the “happy No-Go” condition with the “neutral No-Go” condition to investigate patterns of positive emotional processing within the fronto-thalamic-striatal circuit. The Go/No-Go task assessed response inhibition, comparing happy and neutral No-Go conditions where participants withheld responses to emotional stimuli (Wessa, 2007). We hypothesized the following: (1) adolescents with BD would present enhanced brain activity in neural circuits for cognitive and emotional regulation such as the fronto-thalamic-striatal and fusiform gyrus during emotional response inhibition; and (2) adolescents with BD may exhibit abnormal emotional regulation and difficulties in response inhibition when facing with positive emotional stimuli. This study should help to reveal neural mechanisms underlying emotional regulation difficulties in adolescents with BD. Additionally, it should offer a theoretical foundation for clinical interventions, such as developing therapeutic strategies targeting positive emotional dysregulation.

## 2 Methods

### 2.1 Participants

**Fig. 1:**
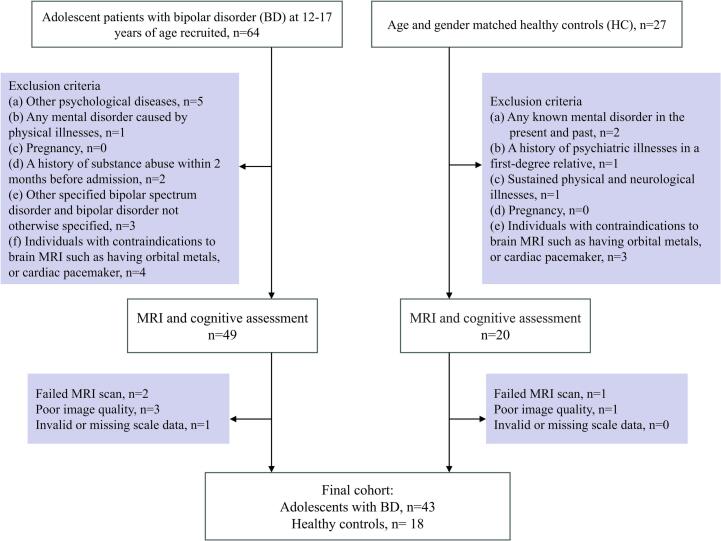
Flow-chart illustrating the enrollment process for adolescents with bipolar disorder (BD) and healthy control (HC) participants.

In the present study, 43 adolescents with BD (mean age=15 years, 21 males) and 18 healthy age- & gender matched healthy controls (HCs) (mean age=14.50 years, 7 males), were recruited. Table 1 presents the detailed clinical demographic information of this cohort. All adolescents with BD were enrolled from the outpatient clinic of the Department of Child and Adolescent Psychiatry at the Second Xiangya Hospital in Changsha, Hunan, P.R. China. During the same study interval, age & gender matched HCs were enrolled from local middle and high schools. The study enrollment process was presented in Fig. 1**.** This study received approval from the ethics committee. All participants and parents signed written informed consent. Inclusion criteria for the adolescents with BD included the following: (1) aged 12 to 17 years fulfilling the Diagnostic and Statistical Manual of Mental Disorders, V Edition (DSM-V) criteria for BD; and (2) able to remain still and follow instructions during fMRI scanning. (3) All patients were right-handed, with no contraindications for fMRI scan. For the HC group, inclusion criteria included the following: age between 12 and 17 years, and no history of psychiatric disorders or psychoactive medication. Exclusion criteria for both groups included the following: (1) presence of other mental disorders, such as autistic spectrum disorder, schizophrenia spectrum disorder, attention deficit and hyperactivity disorder (ADHD) and obsessive-compulsive disorder (OCD); (2) any mental disorder caused by physical illnesses; (3) pregnancy; (4) being left-handed or with contraindication for fMRI scan such as orbital metal or cardiac pacemaker. All participants were required to avoid alcohol or psychoactive substances for at least 24 hours prior to the brain fMRI scan.

**Table 1:** Clinical and demographic characteristics of adolescents with bipolar disorder (BD) with and the healthy controls (HCs).

Group	BD (n=43)	HC (n=18)	*p*-value
Age (years)	15.00 (14.00, 16.00)	14.50 (12.00, 15.00)	0.164
Gender (male/ female)	21/22	7/11	0.477
Onset age (years)	14.00 (13.00, 15.00)		
PSQI	6.00 (3.00, 9.00)	3.00 (2.00, 6.25)	0.022*
MFQ	8.00 (5.00, 19.00)	6.00 (3.50, 8.25)	0.027*
YMRS	6.00 (4.00, 33.00)	3.00 (1.75, 5.25)	＜0.001***
TMT-A	37.00 (29.00, 47.00)	30.00 (24.25, 34.25)	0.008**
TMT-B	81.00 (69.00, 100.00)	72.00 (61.50, 94.25)	0.297
VR	11.00 (8.00, 12.00)	14.00 (13.75, 14.00)	＜0.001***
DST-A	8.00 (7.00, 9.00)	9.00 (8.00, 10.00)	0.031*
DST-B	4.00 (3.00, 6.00)	5.50 (5.00, 8.00)	0.002**
SCWT-A	53.67±14.06	64.89±11.39	0.004**
SCWT-B	72.16±16.07	87.72±9.34	＜0.001***
SCWT-C	31.12±7.99	39.61±8.24	＜0.001***
BP-I/BP-II	28/15		

Notes: Data was presented as median (IQR) or mean ± SD. *p < 0.05, **p < 0.01 and ***p < 0.001 suggest a significant difference between the characteristics in the two cohorts.

Abbreviations: DST, digit span subtest; MFQ, Mood and Feelings Questionnaire; PSQI, Pittsburgh sleep quality index; SCWT, Stroop color-word test; TMT, Trail Making Test; VR, visual reproduction; YMRS, Young Mania Rating Scale.

### 2.2 Procedures

#### 2.2.1 Structured interviews and cognitive assessment

All participants underwent structured interviews conducted by the study child psychiatrists using the Schedule for Affective Disorders and Schizophrenia for School-Age Children Present and Lifetime Version (K-SADS-PL) (Kaufman, 1997). The inter-rater reliability among psychiatrists was satisfactory, with a Kendall’s coefficient of concordance of 0.9. Emotional symptoms of all participants were assessed using the Young Mania Rating Scale (YMRS) (Young, 1978) and the Mood and Feelings Questionnaire (MFQ) (Wood, 1995), while sleep quality was evaluated using the Pittsburgh Sleep Quality Index (PSQI) (Buysse, 1989). In our study, we used cognitive tests that reflected the primary neurocognitive deficits in patients with BD, including attention, memory, and executive function (Solé, 2017). The Stroop Color and Word Test (SCWT) (Scarpina and Tagini, 2017) was used to assess participants’ attention and impulse control. This test consisted of three subtasks: SCWT-A assessed attention by reading characters, SCWT-B evaluated attention by reading colors, and SCWT-C measured impulse control by ignoring color interference. In addition, the Trail Making Test (TMT) (Demakis, 2004) was used to assess attention and processing speed. TMT-A evaluated processing speed and attention, while TMT-B measured cognitive flexibility. We also administered the Digit Span Test (DST) (Wechsler, 1999): DST-A (forward repetition of specified numbers) was used to measure attention, and DST-B (backward repetition of specified numbers) was used to evaluate working memory. Finally, the Visual Reproduction (VR) subtest from the Wechsler Memory Scale-Revised (Hori, 2013) was used to assess visual memory.

#### 2.2.2 Go/No-Go task-based fMRI

**Fig. 2:**
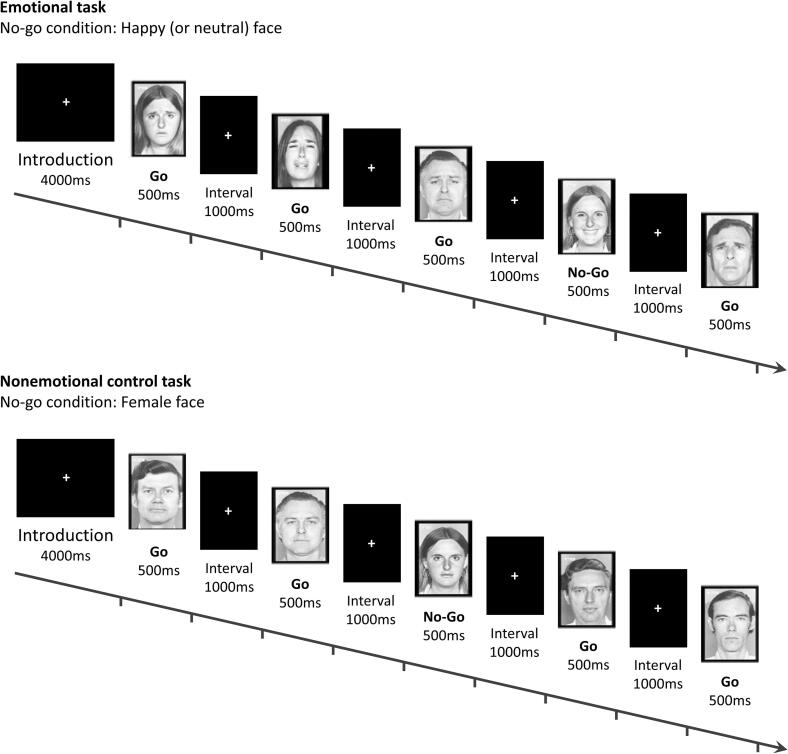
The procedure of an emotional and non-emotional (control) Go/No-Go brain functional MRI (fMRI) paradigm.

The timeline of events of the Emotional Go/No-Go task (Wessa, 2007) is shown in the Fig. 2**.** Participants were trained on the Go/No-Go task prior to the fMRI scanning. They were asked to identify happy, sad, and neutral facial expressions from facial emotion pictures (Ekman and W. V., Pictures of Facial Affect. , 1976) and to perform a button-press task, which involved pressing a button when the target face appeared and withholding a response when the distractor appeared. Participants underwent six emotional Go/No-Go test blocks (including happy, sad, and neutral facial expressions, paired to form six blocks regardless of gender) and two control Go/No-Go blocks (including neutral male targets/female distractors and neutral female targets/male distractors). Each block lasted 36 seconds and contained 16 targets (Go) and 8 distractors (No-Go), which appeared continuously in a pseudo-random sequence. Each facial stimulus was presented for 500 milliseconds, with a stimulus interval of 1,000 milliseconds. All participants were required to read a 4,000-millisecond instruction on the screen before the start of each block. E-prime software was used to record the response time and accuracy of each subject.

The Go/No-Go task was used to measure response inhibition and impulse control (Xia, 2024, Simmonds et al., 2008). Here, we focus primarily on the comparison between the happy No-Go and neutral No-Go conditions, where participants were required to withhold their responses (No-Go) to both happy and neutral facial stimuli. The No-Go condition tested individuals’ ability to inhibit prepotent response tendencies, thereby reflecting their cognitive control ability (Li, 2006, Kiefer, 1998). Happy faces were more representative of real-life social and reward cues, which were particularly important for adolescents. Selecting happy faces as No-Go stimuli allowed us to examine the unique interference pattern of positive emotional arousal as opposed to sad or neutral stimuli on response inhibition control. This should help to assess whether adolescents with BD were more prone to losing control or acting impulsively when confronted with positive emotional triggers.

#### 2.2.3 fMRI acquisition

During the fMRI Go/No-Go task, the stimuli were projected onto a screen by a laptop, and participants viewed the screen through a mirror. Brain fMRI data acquisition and preprocessing MRI images were collected on a 3.0 T Siemens Trio scanner (Siemens, Munich, Germany). Functional images were acquired using a T2-weighted single-echo gradient echo imaging sequence. The scanning parameters were: TR = 2000 milliseconds (ms); TE = 30 ms; field of view (FOV) = 240 mm × 240 mm; flip angle = 90°; matrix size = 64 × 64; slice thickness = 4 mm; and gap = 0.4 mm. The fMRI scan lasted for 620 seconds, yielding a total of 310 volumes. Structural images were acquired using a 3D magnetization-prepared rapid gradient echo (3D MPRAGE) protocol. Detailed parameters for whole-brain coverage included: repetition time (TR) = 2300 ms; echo time (TE) = 2.03 ms; FOV = 256 mm × 256 mm; flip angle = 9°; matrix size = 256 × 256, slice thickness = 1 mm, and gap = 0 mm (Xiao, 2020).

#### 2.2.4 fMRI analysis

Spatial preprocessing of fMRI datasets was conducted using the SPM12 software (http://www.fil.ion.ucl.ac.uk/spm). The fMRI images were corrected for temporal differences between slices and head movement. The fMRI images were realigned with reference to the first scan, spatially normalized using the SPM12 echo planar imaging template and resampled to an iso-voxel size of 3 mm × 3 mm × 3 mm. The images were smoothed by an 8 mm × 8 mm × 8 mm. Gaussian kernel to reduce spatial noise (Wessa, 2007).

The fMRI data from each participant were analyzed using a general linear model with a delayed boxcar waveform to model blood-oxygen level dependent signals in a required condition. The movement parameters computed in the rearrangement process were enrolled in this model as parameters of noninterest in order to remove non-stationary signals because of movement artifacts. For each subject, image differences in the following comparisons of interest were analyzed. Emotional distractors (happy and sad) and neutral distractors were compared to explore the effect of emotional face stimuli on response inhibition in adolescents with BD (Wessa, 2007).

Regions used for brain-behavior analyses were defined from thresholded group-level GLM results. Significant clusters were treated as functionally derived masks, and the mean signal across all voxels within each cluster was extracted for subsequent correlation analyses. Anatomical localization was performed using the AAL atlas. Statistical maps were thresholded at a voxel-level threshold of p < 0.001 (uncorrected). Cluster-level significance was determined using false discovery rate (FDR) correction at p < 0.05, as implemented in SPM12. No additional cluster extent threshold was applied beyond the cluster-level correction.

#### 2.2.5 Statistical analysis

Data were analyzed using SPSS version 27.0 (SPSS, Inc., Chicago, IL, USA). Statistical significance was set at p<0.05. Descriptive statistics, including means and standard deviations, were calculated based on the obtained data, and the Shapiro-Wilk test was used to assess the normality of the distribution of continuous variables. If these variables followed a normal distribution, Student’s t-test was used for group comparisons; otherwise, the Mann-Whitney U test was applied.

The voxelwise T statistics (a two-sample T-test) for fMRI data were created using a second-level, random-effect analysis. The T-map was set at a corrected significance level of *p*<0.05 using the false discovery rate (FDR) criterion (Xiao, 2019).

Statistical analysis of demographic and fMRI performance data was performed using SPSS version 27.0. Independent sample t-tests were used to analyze behavioral performance (reaction time, commission errors = responses to the No-Go trials, and omission errors = failure to respond to the Go trials). Independent sample t-tests and chi-square tests were used to examine group differences in demographic and clinical data. Data were presented as mean±standard deviation (SD). The association between neural activity and behavioral or cognitive measures was examined using multiple linear regression analyses, with age and sex included as covariates. To control for multiple comparisons, a false discovery rate (FDR) correction (Benjamini-Hochberg procedure) was applied separately within each set of tests (i.e., brain-cognition and brain-behavior associations), with a significance threshold set at q<0.05.

## 3 3.Results

### 3.1 Clinical demographic and psychological assessment data

The demographic data, clinical features and psychological assessment data with executive function testing scores in the adolescents with BD and the HCs are presented in Table 1. The adolescents with BD group consisted of 21 males and 22 females, while the HC group consisted of 7 males and 11 females. No significant differences were observed in terms of age and gender between the two groups (*p*=0.477). Regarding mood symptoms, there was a significant difference between the two groups in the mania score (YMRS) (*p*<0.001) and in the mood/feeling score (MFQ) (*p*=0.027). However, the scale scores of VR, DST-A, DST-B, SCWT-A, SCWT-B and SCWT-C in adolescents with BD were significantly lower than those of the HCs (*p*<0.05).

Regarding the behavioral performance data during the fMRI, there were no significant differences in omission errors (emotional target: *p*=0.983; neutral target: *p*=0.964; control target: *p*=0.140), false response errors (emotional distractor: *p*=0.218; neutral distractor: *p*=0.066; control distractor: *p*=0.646) and reaction time (neutral target: *p*=0.118) between the patient group and the HC group, while there were significant differences in reaction time (emotional target: *p*=0.035, control target: *p*=0.042) (Table 2).

**Table 2:** Behavioral performances in an emotional Go/No-Go functional MRI task between the patients with bipolar disorder (BD) and the healthy control (HC) group.

	BD (n=43)	HC (n=18)	*p*-value
Omission errors (%)			
Emotional targets (go)	4.45±1.87	4.44±2.28	0.983
Neutral targets (go)	3.73±1.89	3.70±1.92	0.964
Control targets (go)	2.2±1.3	1.72±1.05	0.14
False response errors (%)			
Emotional distractors (no go)	3.46±1.27	2.99±1.48	0.218
Neutral distractors (no go)	2.95±1.56	2.36±0.88	0.066
Control distractors (no go)	2.75±1.08	2.60±1.22	0.646
Reaction time (millisecond)			
Emotional targets (go)	635.17±95.63	589.77±63.57	0.035*
Neutral targets (go)	624.79±85.79	588.85±66.41	0.118
Control targets (go)	597.63±85.08	551.27±63.21	0.042*

Notes: Data were expressed as mean ± SD.

### 3.2 fMRI neuroimaging data

#### 3.2.1 Happy No-Go versus neutral No-Go conditions

**Fig. 3:**
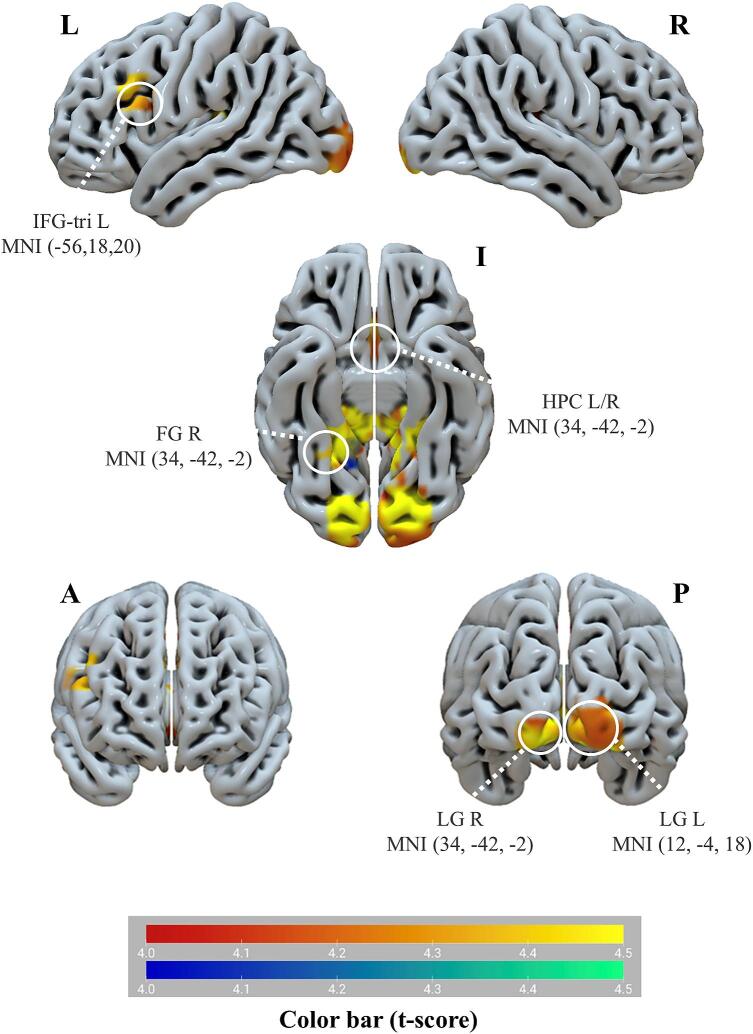
Activity in brain regions of adolescents with bipolar disorder (BD) as compared to the healthy controls. Note: Warm colors (red to yellow) indicate regions with increased activity and cool colors (blue to green) indicate regions with decreased activity. Abbreviations: L, left hemisphere; R, right hemisphere; I, inferior view; P, posterior view; A, anterior view; IFG-tri, inferior frontal gyrus, triangular part; FG, fusiform gyrus; HPC, hippocampus; LG, lingual gyrus.

The patient group showed a significant cluster of activations during the contrast happy > neutral distractors in the left inferior frontal gyrus (triangular part), the right fusiform gyrus, the left cerebellum Crus I, the right caudate nucleus, the left caudate nucleus, the right cerebellum VI, the left fusiform gyrus, the right lingual gyrus, the right thalamus, the right hippocampus, the right parahippocampal gyrus, the left lingual gyrus, the left thalamus, the left hippocampus and the left parahippocampal gyrus, relative to the HC group (*p*<0.05, FDR corrected) (Table 3 **and** Fig. 3). Gender effect was not identified when comparing all emotional and neutral Go/No-Go conditions.

**Table 3:** Altered activity of brain regions in the adolescents with bipolar disorder (BD) group and the healthy control (HC) group from comparisons of response inhibition to emotional versus neutral distractors (false discovery rate (FDR) corrected).

Brain regions	Hemisphere	AAL	MNI Peak coordinates			T-value	Voxel number
			X	Y	Z		
Cerebellum Crus I	L	91	-20	-82	-28	4.09	12
Cerebellum VI	R	100	40	-46	-24	4.23	21
Fusiform gyrus	R	56	40	-46	-24	4.23	21
	R	56	34	-42	-2	5.13	290
	L	55	12	-4	18	4.23	550
Inferior frontal gyrus, triangular part	L	13	-56	18	20	4.47	6
Caudate nucleus	R	72	12	-4	18	4.23	550
	L	71					
Lingual gyrus	R	48	34	-42	-2	5.13	290
Thalamus		78					
Hippocampus		38					
Parahippocampal gyrus		40					
Lingual gyrus	L	47	12	-4	18	4.23	550
Thalamus		77					
Hippocampus		37					
Parahippocampal gyrus		39					

Abbreviations: AAL, anatomical automatic labeling; Brodmann Area; L, left; MNI, Montreal Neurological Institute; R, right.

### 3.3 Correlations between neural activity and cognitive testing scores

**Fig. 4:**
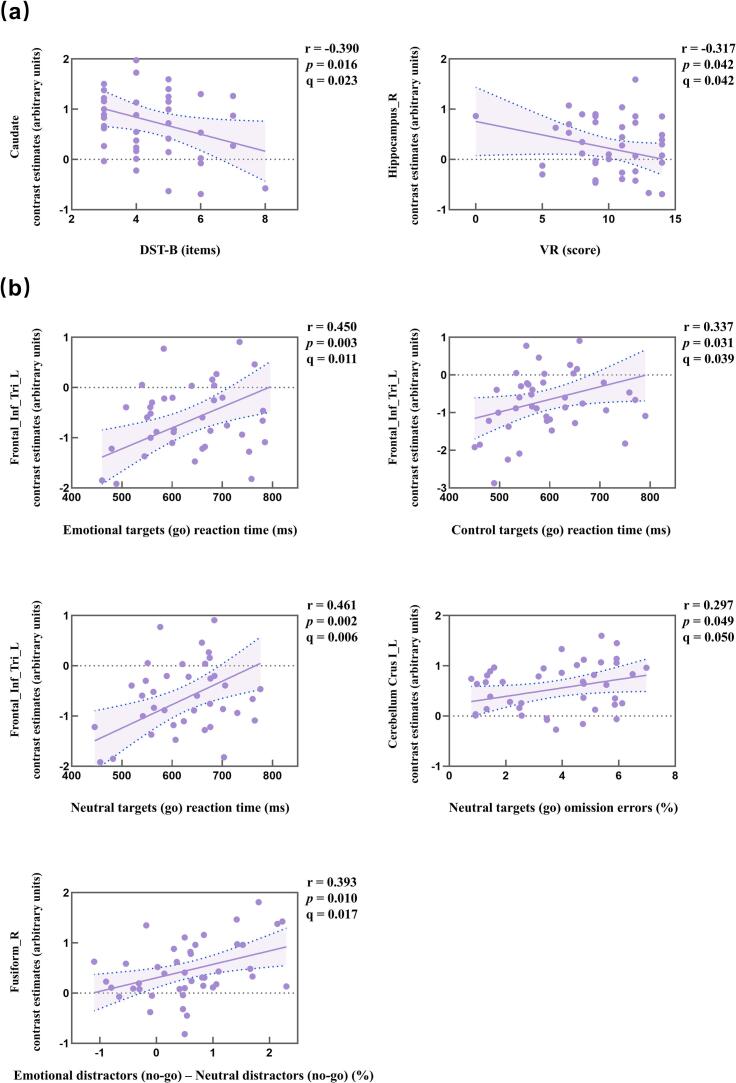
Correlations of brain activity with cognitive assessment scores and false response errors from the Go/No-Go functional brain MRI. **a.** Negative correlation between the brain activity in response to task-relevant positive versus neutral distractors with cognitive assessment scores. **b.** Positive correlation between the brain activity in response to task-relevant happy versus neutral distractors and false response errors from the Go/No-Go functional brain MRI. Abbreviations: L, left; R, right; DST, digit span subtest; VR, visual reproduction; Frontal_Inf_Tri, inferior frontal gyrus, triangular part. Notes: The y‑axes represent contrast estimates in arbitrary units for all displayed brain regions.

The caudate nucleus activity in response to task-relevant happy versus neutral distractors was negatively associated with DST-B in the patient group (r=-0.390, *p*=0.016, q=0.023). The right hippocampus activity in response to task-relevant happy versus neutral distractors was negatively associated with the VR in the patient group (r=-0.317, *p*=0.042, q=0.042) (Fig. 4**a and supplementary file (Fig. S1. A)**). Supplementary file (**Table S1**) provides detailed information about the brain regions included in each cluster shown in the supplementary file (**Fig. S1. A**).

### 3.4 Correlations between neural activity and behavioral task performance data

The left inferior frontal gyrus (triangular part) activity in response to task-relevant happy versus neutral distractors was positively associated with reaction time (emotional target: r=0.45, *p*=0.003, q=0.011, neutral target: r=0.461, *p*=0.002, q=0.006, control target: r=0.337, *p*=0.031, q=0.039) in the patient group. The left cerebellum crus I activity in response to task-relevant happy versus neutral distractors was positively associated with omission errors (neutral target: r=0.297, *p*=0.049, q=0.05) in the patient group. The right fusiform gyrus activity in response to task-relevant happy versus neutral distractors was positively associated with false response errors (r=0.393, *p*=0.01, q=0.017) in the patient group (Fig. 4**b and supplementary file (Fig. S1. B**)). Supplementary file (**Table S1**) provides detailed information about the brain regions included in each cluster shown in the supplementary file (**Fig. S1. B**).

## 4 Discussion

This study showed altered activity in a fronto-thalamic-striatal circuit as well as in the fusiform gyrus, the cerebellum and the hippocampus in adolescents with BD during the processing of happy versus neutral distractor in an emotional Go/No-Go fMRI task. To the best of our knowledge, this was the first study showing abnormal neural responses during the inhibition of positive stimuli in adolescents with BD, which was correlated with cognitive assessment scores and behavioral task performance data.

This study showed alteration of fronto-striatal-thalamic circuit to positive emotional responses including the interaction of the frontal and striatal regions to regulate emotions, reward and impulses in adolescents with BD, similar to literature (Szily and Kéri, 2008). Dysregulation of striatal regions and abnormal functioning of the prefrontal cortex constitute a core component of the neurobiology of BD (Strakowski, 2012, Strakowski, 2016). Our study result was also consistent with a prior fMRI study under emotional Go/No-Go condition showing increased caudate activity in the prefrontal-limbic regions of patients with BD (Wessa, 2007). The caudate nucleus may be over-activated in the manic state, leading to an exaggerated response to positive emotions and rewarding stimuli. The caudate nucleus is well-known for its role in higher-order motor control, recent learning and memory, reward and particularly feedback processing (Packard and Knowlton, 2002). Our observation of caudate nucleus activity being negatively correlated with cognitive testing scores such as the DST scores indicated its potential role in cognitive functioning in patients with BD. However, another study reported no significant differences in response to happy facial stimuli between patients with BD and the controls (Lennox, 2004), which was not in agreement with our results. The discrepancy between our and their results may partly due to their cohort consisting patients all with bipolar I mania while our cohort was more representative of patients with BD having patients with both mania and depression.

Our study showed that increased activation in the caudate nucleus, thalamus, and inferior frontal gyrus in adolescents with BD as compared to the HCs. These brain regions are components of the prefrontal-striatal-thalamic circuit, and dysfunction in this circuit has been linked to the pathophysiology of BD (Wessa, 2007, Blumberg, 2003). Moreover, a prior study has found that patients with BD exhibited abnormally heightened neural activation in the prefrontal-striatal circuit under reward-related conditions indicating a heightened sensitivity to positive stimuli and an overactive response (Nusslock et al., 2014). Additionally, a fMRI study using cognitive tasks demonstrated increased thalamic activity in young patients with BD (Blumberg, 2003). These observations suggested that adolescents with BD may recruit additional cognitive resources to inhibit impulsive responses when exposed to happy faces, reflecting a potential compensatory mechanism for emotional regulation.

This study found increased activity of left inferior frontal gyrus in adolescents with BD, indicating that patients exert more effort in managing emotions as compared to the controls. This finding aligns with a prior study which reported increased left inferior frontal gyrus activation during an emotional Stroop task involving positive versus neutral words in patients with BD as compared to the controls (Passarotti et al., 2010). As part of the fronto-striatal-thalamic circuit, the inferior frontal gyrus plays a critical role in emotional regulation. However, conflicting results were reported in other studies (Lagopoulos and Malhi, 2007, Malhi, 2005, Pavuluri, 2008), possibly due to differences in disease severity. In mild cases, dysfunction of emotional regulation circuit may manifest as reduced activation (Malhi, 2005); whereas in more severe cases, patients may need additional effort in emotional regulation, leading to compensatory hyperactivation (Pavuluri, 2008). In addition; our study was consistent with a prior study showing adolescent patients with BD with significantly slower reaction times than the HCs during a positive-versus-neutral word comparison (Passarotti et al., 2010). Furthermore, previous studies have shown that patients with BD may exhibit attention and cognitive control deficits (Dickstein, 2005, Pavuluri, 2009, Pavuluri, 2006), and our results corroborated it. Increased left inferior frontal gyrus activation was associated with longer Go reaction times, suggesting poorer attention. Our study results implicated the dual role of inferior frontal gyrus in the pathophysiology of adolescents with BD, i.e., its hyperactivation reflecting compensatory involvement in emotional regulation, and its contribution to attentional control deficits.

Our study also found increased hippocampal activity in adolescents with BD, which was negatively correlated with the visual memory function reflecting cognitive impairment. As part of the limbic system, the hippocampus is involved in the regulation, expression, and memory of emotions. A fMRI study using emotional face stimuli found increased activation in the amygdala, hippocampus, and orbitofrontal cortex in patients with BD during both manic and remission episodes as compared to the controls (Chen, 2010). This was largely in agreement with our findings, though their study did not specify specific emotions but compared emotional faces to neutral ones. Our study specifically focused on happy faces suggested that positive emotions may play a prominent role affecting patients’ emotional status. Additionally, a machine-learning study (Lei, 2023) found that baseline functional activity in the limbic system in patients with BD could predict treatment outcomes for different medications, suggesting its potential as a biomarker for treatment decision-making and for clinical prognosis.

In this study, the enhanced activity of the fusiform gyrus may indicate that patients were more sensitive to the processing of happy faces or had an exaggerated response to positive emotional stimuli. The fusiform gyrus is part of the visual network and is involved in face recognition, with a specific region (FFA, fusiform face area) responsible for processing faces and complex visual stimuli (Kanwisher et al., 1997). Therefore, during the execution of the happy No-Go task, the patients found it more challenging to suppress their responses, leading to a higher error rate as shown in this study. This result suggested that the increased activity in this specific brain regions when processing positive emotional stimuli may reflect a higher emotional arousal level elicited by happy faces, which made it more challenging for them to inhibit their responses. In addition, recent studies have shown that the fusiform gyrus also plays a role in the default mode network, which is associated with information processing and memory, further affecting response to positive stimuli. This network is typically active during rest and suppressed during cognitive tasks (Menon, 2023). An fMRI study found that certain brain regions of the default network exhibit coordinated activity patterns with the visual-related network during both resting states and visual processing tasks (Greicius, 2003). This suggested that when the visual network was active, it may simultaneously activate the default mode network, leading to task interference and resulting in errors. In this context, the errors made by the patients during the fMRI Go/No-Go may be due to the activation of the visual-related network and coordinated activation of the default mode network, leading to impulsive responses.

The adolescent patients with BD in this study also exhibited enhanced activity in the left cerebellum Crus I under the happy No-Go versus neutral No-Go condition as compared to the controls. The cerebellum is closely associated with higher cognitive functions and emotional regulation (Stoodley and Schmahmann, 2009). In the No-Go task, cerebellar activity was positively correlated with omission errors, meaning that stronger activity was associated with higher omission rates. This result may indicate that patients require greater cognitive resources to suppress impulsive responses when facing happy faces. In other words, it is more challenging for adolescent patients with BD to inhibit impulses when exposed to happy faces, making them more prone to errors during the No-Go task.

There were several limitations to this study. First, this study was limited by its modest sample size and lack of longitudinal data as we were constrained by the challenges of recruiting adolescent patients and conducting follow-up assessments including fMRI scans. Second, due to the limited sample size, we could not perform additional analysis distinguishing between bipolar subtypes or mood states, which could potentially generate more relevant data regarding specific type of BD. Future studies with larger samples and longitudinal data are needed and are being planned to validate the results from this study. Third, all patients with BD in this cohort had a history of psychotropic medications (e.g., mood stabilizers like lithium, antipsychotics such as chlorpromazine and olanzapine, and antidepressants such as sertraline and paroxetine), which could potentially lead to cognitive or memory-related changes, inattention, and abnormal brain function. It was not clear as whether these medications had an overall impact on the study results. However, previous studies suggest that common neural activity changes caused by these medications tend to correct the original abnormalities and reduce overactivity (Dale, 2016, López-Jaramillo, 2017), rather than exacerbate them, thus potentially masking disease-related neural abnormalities to some extent. Therefore, the abnormally enhanced activity observed during the fMRI exam for this study was unlikely to be attributable to the medications and thus could be due to the disorder itself. Furthermore, we implemented measures to control for medication effects for this study, such as excluding participants with recent medication changes or significant dose adjustments, and ensuring participants with stable medication doses for at least two months prior to enrollment. Finally, regions showing significant group differences were subsequently used for correlation analyses with behavioral and cognitive measures within the same dataset. This approach may have introduced non-independence (“double dipping”) and may have inflated effect sizes. Therefore, these findings should be considered exploratory. Future studies using independent datasets, cross-validation strategies, or a priori defined regions of interest are needed to confirm the robustness of these associations. In addition, multiple correlation analyses were performed across several regions and behavioral/cognitive measures. Although FDR correction was applied, the number of tests may have increased the risk of false-positive findings. Therefore, these associations should be interpreted with caution and should be considered exploratory until replicated in independent samples.

## 5 Conclusion

In summary, our study identified abnormal neural responses to positive stimuli and their correlations with cognitive impairment in adolescents with BD, contributing to a deeper understanding of the neurobiological mechanism underlying adolescent BD. Specifically, the characteristic fMRI changes in emotional processing and inhibitory control under happy face stimuli may serve as potential neuroimaging correlates of this disorder in vulnerable adolescents.

**Data availability statement:** Research data are not shared.

**Ethical approval statement:** This research was approved by the Ethics Committee and Institutional Review Board in our hospital (IRB No. 2022020227).

**Patient consent statement:** Written informed consent for the adolescents in this study were obtained from their parents or legal guardians.

## CRediT authorship contribution statement

**Xueying Wang:** Writing – review & editing, Writing – original draft, Validation, Methodology, Formal analysis, Conceptualization. **Jinfan Zhang:** Project administration, Investigation. **Feifei Wu:** Project administration, Investigation. **Liying Shen:** Project administration, Investigation. **Lin Wang:** Project administration, Investigation. **Han Wu:** Project administration. **Qian Xiao:** Writing – review & editing, Supervision, Funding acquisition, Data curation, Conceptualization. **Xiaoping Yi:** Writing – review & editing, Methodology, Data curation, Conceptualization. **Xiaoqun Liu:** Resources, Project administration, Data curation. **Alessandro Grecucci:** Writing – review & editing. **Bihong T. Chen:** Writing – review & editing.

## Funding

This research was funded in part by the Youth Fund of National Natural Science Foundation of China (grant No.82201702), Natural Science Foundation of Hunan Province (grant No.2025JJ50591), Scientific Research Program of FuRong Laboratory (grant No.2024PT5109) and Fundamental Research Funds for the Central Universities of Central South University (grant 2024ZZTS0954).

## Declaration of competing interest

The authors declare that they have no known competing financial interests or personal relationships that could have appeared to influence the work reported in this paper.

## Data Availability

The authors do not have permission to share data.

## Appendix A Supplementary data

Supplementary data 1 Figure S1. Associations between brain activations and cognitive/task performance measures. Table S1. Brain regions included in clusters presented in Figure S1.

## References

[b0005] Müller J.K., Leweke F.M. (2016). *Bipolar disorder: clinical overview*. Med Monatsschr Pharm.

[b0010] McClellan J., Werry J. (1997). *Practice parameters for the assessment and treatment of children and adolescents with bipolar disorder. American Academy of Child and Adolescent Psychiatry*. J Am Acad Child Adolesc Psychiatry.

[b0015] Hirshfeld-Becker D.R. (2003). *Behavioral inhibition and disinhibition as hypothesized precursors to psychopathology: implications for pediatric bipolar disorder*. Biol Psychiatry.

[b0020] Lawlor-Savage L., Sponheim S.R., Goghari V.M. (2014). *Impaired recognition of happy facial expressions in bipolar disorder*. Acta Neuropsychiatr.

[b0025] Frías Á., Palma C., Farriols N. (2014). *Neurocognitive impairments among youth with pediatric bipolar disorder: a systematic review of neuropsychological research*. J Affect Disord.

[b0030] Elias L.R. (2017). *Cognitive Impairment in Euthymic Pediatric Bipolar Disorder: A Systematic Review and Meta-Analysis*. J Am Acad Child Adolesc Psychiatry.

[b0035] Crone E.A., Dahl R.E. (2012). *Understanding adolescence as a period of social-affective engagement and goal flexibility*. Nat Rev Neurosci.

[b0040] Galvan A. (2006). *Earlier development of the accumbens relative to orbitofrontal cortex might underlie risk-taking behavior in adolescents*. J Neurosci.

[b0045] Somerville L.H., Casey B.J. (2010). *Developmental neurobiology of cognitive control and motivational systems*. Curr Opin Neurobiol.

[b0050] Hafeman D.M. (2014). *Abnormal deactivation of the inferior frontal gyrus during implicit emotion processing in youth with bipolar disorder: attenuated by medication*. J Psychiatr Res.

[b0055] Logothetis N.K. (2001). *Neurophysiological investigation of the basis of the fMRI signal*. Nature.

[b0060] Breakspear M. (2015). *Network dysfunction of emotional and cognitive processes in those at genetic risk of bipolar disorder*. Brain.

[b0065] Delvecchio G., Sugranyes G., Frangou S. (2013). *Evidence of diagnostic specificity in the neural correlates of facial affect processing in bipolar disorder and schizophrenia: a meta-analysis of functional imaging studies*. Psychol Med.

[b0070] Mesbah R. (2023). *Association Between the Fronto-Limbic Network and Cognitive and Emotional Functioning in Individuals With Bipolar Disorder: A Systematic Review and Meta-analysis*. JAMA Psychiatry.

[b0075] Strakowski S.M. (2012). *The functional neuroanatomy of bipolar disorder: a consensus model*. Bipolar Disord.

[b0080] Rich B.A. (2008). *Neural connectivity in children with bipolar disorder: impairment in the face emotion processing circuit*. J Child Psychol Psychiatry.

[b0085] Ladouceur, C.D., et al., Differential patterns of abnormal activity and connectivity in the amygdala-prefrontal circuitry in bipolar-I and bipolar-NOS youth. J Am Acad Child Adolesc Psychiatry, 2011. 50(12): p. 1275-89.e2. 10.1016/j.jaac.2011.09.023 PMC3268077 22115148

[b0090] Morris R.W. (2012). *Lack of cortico-limbic coupling in bipolar disorder and schizophrenia during emotion regulation*. Transl Psychiatry.

[b0095] Passarotti A.M. (2012). *Reduced functional connectivity of prefrontal regions and amygdala within affect and working memory networks in pediatric bipolar disorder*. Brain Connect.

[b0100] Toma S. (2019). *Cortical Volume and Thickness Across Bipolar Disorder Subtypes in Adolescents: A Preliminary Study*. J Child Adolesc Psychopharmacol.

[b0105] Haber S.N., Knutson B. (2010). *The reward circuit: linking primate anatomy and human imaging*. Neuropsychopharmacology.

[b0110] Péron J. (2017). *Vocal emotion decoding in the subthalamic nucleus: An intracranial ERP study in Parkinson's disease*. Brain Lang.

[b0115] Brown E.S. (2007). *Effects of chronic prednisone therapy on mood and memory*. J Affect Disord.

[b0120] Congdon E. (2010). *Engagement of large-scale networks is related to individual differences in inhibitory control*. Neuroimage.

[b0125] Lapomarda G. (2021). *Out of control: An altered parieto-occipital-cerebellar network for impulsivity in bipolar disorder*. Behav Brain Res.

[b0130] Lapomarda G. (2021). *Common and different gray and white matter alterations in bipolar and borderline personality disorder: A source-based morphometry study*. Brain Res.

[b0135] Strakowski S.M., Delbello M.P., Adler C.M. (2005). *The functional neuroanatomy of bipolar disorder: a review of neuroimaging findings*. Mol Psychiatry.

[b0140] Hwang J. (2006). *Basal ganglia shape alterations in bipolar disorder*. Am J Psychiatry.

[b0145] Rich B.A. (2010). *A preliminary study of the neural mechanisms of frustration in pediatric bipolar disorder using magnetoencephalography*. Depress Anxiety.

[b0150] Wessa M. (2007). *Fronto-striatal overactivation in euthymic bipolar patients during an emotional go/nogo task*. Am J Psychiatry.

[b0155] Kaufman J. (1997). *Schedule for Affective Disorders and Schizophrenia for School-Age Children-Present and Lifetime Version (K-SADS-PL): initial reliability and validity data*. J Am Acad Child Adolesc Psychiatry.

[b0160] Young R.C. (1978). *A rating scale for mania: reliability, validity and sensitivity*. Br J Psychiatry.

[b0165] Wood A. (1995). *Properties of the mood and feelings questionnaire in adolescent psychiatric outpatients: a research note*. J Child Psychol Psychiatry.

[b0170] Buysse D.J. (1989). *The Pittsburgh Sleep Quality Index: a new instrument for psychiatric practice and research*. Psychiatry Res.

[b0175] Solé B. (2017). *Cognitive Impairment in Bipolar Disorder: Treatment and Prevention Strategies*. Int J Neuropsychopharmacol.

[b0180] Scarpina F., Tagini S. (2017). *The Stroop Color and Word Test*. Front Psychol.

[b0185] Demakis G.J. (2004). *Frontal lobe damage and tests of executive processing: a meta-analysis of the category test, stroop test, and trail-making test*. J Clin Exp Neuropsychol.

[b0190] Wechsler D. (1999).

[b0195] Hori T. (2013). *Visual reproduction on the Wechsler Memory Scale-Revised as a predictor of Alzheimer's disease in Japanese patients with mild cognitive impairments*. Dement Geriatr Cogn Disord.

[b0200] Ekman, P.F., W. V., *Pictures of Facial Affect*. 1976, Palo Alto, CA: Consulting Psychologists Press.

[b0205] Xia Y. (2024). *Impulsivity and neural correlates of response inhibition in bipolar disorder and their unaffected relatives: A MEG study*. J Affect Disord.

[b0210] Simmonds D.J., Pekar J.J., Mostofsky S.H. (2008). *Meta-analysis of Go/No-go tasks demonstrating that fMRI activation associated with response inhibition is task-dependent*. Neuropsychologia.

[b0215] Li C.S. (2006). *Imaging response inhibition in a stop-signal task: neural correlates independent of signal monitoring and post-response processing*. J Neurosci.

[b0220] Kiefer M. (1998). *The time course of brain activations during response inhibition: evidence from event-related potentials in a go/no go task*. Neuroreport.

[b0225] Xiao Q. (2020). *Gray matter voxel-based morphometry in mania and remission states of children with bipolar disorder*. J Affect Disord.

[b0230] Xiao Q. (2019). *Altered regional homogeneity in pediatric bipolar disorder during manic and euthymic state: a resting-state fMRI study*. Brain Imaging Behav.

[b0235] Szily E., Kéri S. (2008). *Emotion-related brain regions*. Ideggyogy Sz.

[b0240] Strakowski S.M. (2016). *fMRI brain activation changes following treatment of a first bipolar manic episode*. Bipolar Disord.

[b0245] Packard M.G., Knowlton B.J. (2002). *Learning and memory functions of the Basal Ganglia*. Annu Rev Neurosci.

[b0250] Lennox B.R. (2004). *Behavioural and neurocognitive responses to sad facial affect are attenuated in patients with mania*. Psychol Med.

[b0255] Blumberg H.P. (2003). *Frontostriatal abnormalities in adolescents with bipolar disorder: preliminary observations from functional MRI*. Am J Psychiatry.

[b0260] Nusslock R., Young C.B., Damme K.S. (2014). *Elevated reward-related neural activation as a unique biological marker of bipolar disorder: assessment and treatment implications*. Behav Res Ther.

[b0265] Passarotti A.M., Sweeney J.A., Pavuluri M.N. (2010). *Differential engagement of cognitive and affective neural systems in pediatric bipolar disorder and attention deficit hyperactivity disorder*. J Int Neuropsychol Soc.

[b0270] Lagopoulos J., Malhi G.S. (2007). *A functional magnetic resonance imaging study of emotional Stroop in euthymic bipolar disorder*. Neuroreport.

[b0275] Malhi G.S. (2005). *An emotional Stroop functional MRI study of euthymic bipolar disorder*. Bipolar Disord.

[b0280] Pavuluri M.N. (2008). *An fMRI study of the interface between affective and cognitive neural circuitry in pediatric bipolar disorder*. Psychiatry Res.

[b0285] Dickstein D.P. (2005). *Neurologic examination abnormalities in children with bipolar disorder or attention-deficit/hyperactivity disorder*. Biol Psychiatry.

[b0290] Pavuluri M.N. (2009). *An fMRI study of the neural correlates of incidental versus directed emotion processing in pediatric bipolar disorder*. J Am Acad Child Adolesc Psychiatry.

[b0295] Pavuluri M.N. (2006). *Neurocognitive function in unmedicated manic and medicated euthymic pediatric bipolar patients*. Am J Psychiatry.

[b0300] Chen C.H. (2010). *A longitudinal fMRI study of the manic and euthymic states of bipolar disorder*. Bipolar Disord.

[b0305] Lei D. (2023). *Effects of short-term quetiapine and lithium therapy for acute manic or mixed episodes on the limbic system and emotion regulation circuitry in youth with bipolar disorder*. Neuropsychopharmacology.

[b0310] Kanwisher N., McDermott J., Chun M.M. (1997). *The fusiform face area: a module in human extrastriate cortex specialized for face perception*. J Neurosci.

[b0315] Menon V. (2023). *20 years of the default mode network: A review and synthesis*. Neuron.

[b0320] Greicius M.D. (2003). *Functional connectivity in the resting brain: a network analysis of the default mode hypothesis*. Proc Natl Acad Sci U S A.

[b0325] Stoodley C.J., Schmahmann J.D. (2009). *Functional topography in the human cerebellum: a meta-analysis of neuroimaging studies*. Neuroimage.

[b0330] Dale E. (2016). *Effects of serotonin in the hippocampus: how SSRIs and multimodal antidepressants might regulate pyramidal cell function*. CNS Spectr.

[b0335] López-Jaramillo C. (2017). *Increased hippocampal, thalamus and amygdala volume in long-term lithium-treated bipolar I disorder patients compared with unmedicated patients and healthy subjects*. Bipolar Disord.

