# Supplementary material for: Alteration of fronto-thalamic-striatal and visual network activity to positive emotional stimuli in adolescent patients with bipolar disorder during a Go/No-Go task-based functional brain MRI
Source: Neuroimage Clin. 2026 May 10;50:104005. doi: 10.1016/j.nicl.2026.104005 (PMC13199762; doi:10.1016/j.nicl.2026.104005)
Supplement: Supplementary data 1 — Figure S1. Associations between brain activations and cognitive/task performance measures. Table S1. Brain regions included in clusters presented in Figure S1. [file mmc1.docx]

**Supplementary files:**


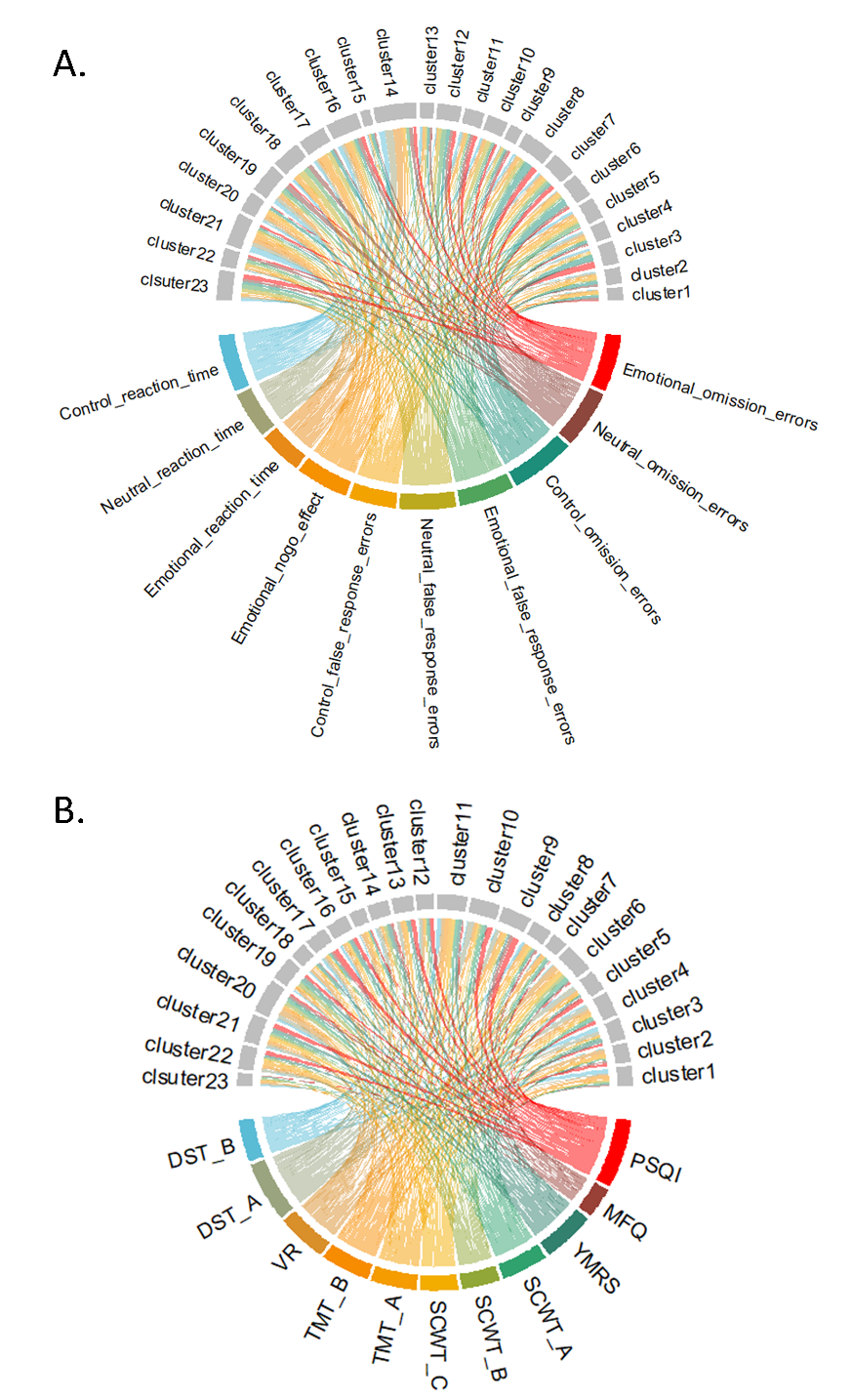


**Fig. S1.** Chord diagrams showing the interrelationship of activations of specific brain regions with task performances and cognitive assessment scores: task performance (FigS1.A) and cognitive assessment scores (FigS1.B). Details of the brain regions included in each cluster are provided in Table S1.

Abbreviations: DST, digit span subtest; TMT, Trail Making Test; VR, visual reproduction; PSQI, Pittsburgh sleep quality index; MFQ, Mood and Feelings Questionnaire; SCWT, Stroop color-word test; YMRS, Young Mania Rating Scale.

| Table S1. Brain regions included in each cluster for Figure S1. | |
| --- | --- |
| **Cluster** | **Brain Region** |
| 1 | Caudate nucleus_R |
|  | Caudate nucleus_L |
|  | Thalamus_L |
|  | Precuneus_L |
|  | Hippocampus_L |
|  | Parahippocampal Gyrus_L |
|  | Lingual Gyrus_L |
|  | Fusiform Gyrus_L |
| 2 | Parahippocampal Gyrus_R |
|  | Fusiform Gyrus_R |
|  | Hippocampus_R |
|  | Lingual Gyrus_R |
|  | Thalamus_R |
|  | Caudate Nucleus_R |
| 3 | Cerebelum_Crus2_L |
|  | Cerebelum_Crus1_L |
|  | Lingual Gyrus_L |
|  | Pericalcarine Cortex_L |
|  | Inferior Occipital Gyrus_L |
|  | Cerebellum_6_L |
|  | Middle Occipital Gyrus _L |
|  | Fusiform Gyrus_L |
| 4 | Cerebelum_6_R |
|  | Vermis_6 |
|  | Vermis_7 |
|  | Vermis_8 |
| 5 | Lingual Gyrus_R |
|  | Cerebelum_Crus1_R |
|  | Fusiform gyrus_R |
|  | Cerebelum_6_R |
|  | Pericalcarine Cortex_R |
|  | Inferior Occipital Gyrus_R |
| 6 | Caudate nucleus_R |
| 7 | Caudate nucleus_R |
|  | Caudate nucleus_L |
|  | Olfactory Cortex_R |
| 8 | Cerebelum_Crus1_R |
|  | Cerebelum_6_R |
|  | Vermis_7 |
|  | Cerebelum_Crus2_R |
| 9 | Caudate nucleus_R |
| 10 | Vermis_4_5 |
|  | Vermis_3 |
|  | Cerebelum_4_5_L |
|  | Cerebelum_3_L |
| 11 | Central Operculum_R |
|  | Inferior Frontal Gyrus, Opercular Part_R |
| 12 | Cerebelum_7b_L |
|  | Cerebelum_Crus2_L |
| 13 | Medial and Paracingulate Gyri_R |
|  | Medial and Paracingulate Gyri_L |
|  | Medial Superior Frontal Gyrus_L |
| 14 | Inferior Frontal Gyrus, Triangular Part_L |
| 15 | Cerebelum_7b_L |
| 16 | Supplementary Motor Area_L |
|  | Medial Superior Frontal Gyrus_L |
| 17 | Fusiform Gyrus_R |
|  | Cerebelum_6_R |
| 18 | Cerebelum_Crus2_L |
| 19 | Cerebelum_Crus1_L |
| 20 | Medial and Paracingulate Gyri_L |
|  | Supplementary Motor Area_L |
| 21 | Cerebelum_Crus1_L |
| 22 | Lingual Gyrus_L |
|  | Vermis_4_5 |
|  | Pericalcarine Cortex_L |
| 23 | Middle Temporal Gyrus_R |

Notes: R, right; L, left.
